# Aggressive tumors and difficult choices in acromegaly

**DOI:** 10.1007/s11102-013-0538-5

**Published:** 2013-12-01

**Authors:** Carmen A. Carrasco, Mônica Gadelha, Marcos Manavela, Oscar D. Bruno

**Affiliations:** 1 Departamento de Endocrinología, Facultad de Medicina, Pontificia Universidad Católica de Chile, Santiago, Chile; 2 Endocrinology Section, Hospital Universitário Clementino Fraga Filho, Federal University of Rio de Janeiro, Rio de Janeiro, Brazil; 3 Division of Endocrinology, Hospital de Clínicas, University of Buenos Aires, Av. Córdoba 2351, 1120 Buenos Aires, Argentina

**Keywords:** Acromegaly, GH, IGF-1, Aggressive tumors, Somatostatin analogs, Dopamine agonists

## Abstract

**Purpose:**

The current article looks at some of the factors associated with pituitary adenomas displaying unusually aggressive biological and clinical behaviour in patients with acromegaly.

**Methods:**

This was a retrospective, narrative review of previously published evidence chosen at the authors’ discretion and presented from the perspective of a Latin American case study.

**Findings and Conclusions:**

Although most pituitary tumors in acromegalic patients are benign and non-aggressive many can behave more aggressively, compromising local surrounding structures. These lesions tend to respond poorly to somatostatin analogs, have a higher risk of recurrence after surgery and, thus, a worse prognosis. Patients with more aggressive tumors constitute a particular challenge, as they often require several therapeutic approaches and may be difficult to manage, especially when options are restricted due to limited resources.

## Introduction

Most pituitary tumors in acromegalic patients are benign and respond well to multimodal therapy [1]. Nevertheless, many pituitary adenomas can behave in a more aggressive manner, with more rapid growth, local invasion into the surrounding structures, greater risk of recurrence after surgery and worse prognosis [1–3]. Patients with invasive and more aggressive GH-secreting tumors often require several therapeutic approaches and may be difficult to manage [1], especially when options are restricted due to limited resources. The current article presents a case report of a somatotropic tumor with aggressive behavior and discusses some of the treatment challenges and difficult choices that this can impose in Latin America.

## What defines an aggressive tumor in acromegaly?

Tumor aggressiveness can be based on clinical, radiological and pathological features. However, there are no well-defined absolute criteria for defining an aggressive pituitary tumor. The 2004 World Health Organization classification of pituitary tumors defined three primary types—“typical pituitary adenoma”, “atypical pituitary adenoma” and “pituitary carcinoma” [4, 5]. The rather poorly defined “atypical” designation was an attempt to identify those adenomas that are likely to display more aggressive behavior with increased proliferative activity. The specific “atypical” characteristics that were identified included invasion into the cavernous sinuses, an elevated proliferation index [Ki-67 labeling index (LI) >3 %] and nuclear reactivity for p. 53 [4]. However, the true value of these and other biomarkers in predicting aggressive and progressive tumor behavior in clinical practice remains uncertain [6, 7]. Many studies have found strong associations between Ki-67 labeling and tumor invasiveness, tumor size and recurrence, but there is inconsistency in the data, which may, at least in part, relate to the different criteria and measures used to define factors such as invasiveness [8]. Histologic subtyping may be the best predictor of aggressive behavior once surgical biopsies have been taken, particularly with regards to the presence of sparse granulation in somatotroph (i.e., growth hormone-secreting) adenomas, although subtyping into sparse versus dense granulation does not absolutely predict aggressiveness and many biopsies may show intermediate characteristics [7, 9, 11].

Radiological assessment of gross tumor invasiveness may be more clinically useful, especially as it can also provide an initial assessment of aggressive behavior obviating the need for intraoperative biopsies. At the time of acromegaly diagnosis, macroadenomas (tumors >10 mm in diameter) represent approximately 80 % of GH secreting adenomas [12–14]. Although definitions of invasiveness vary, approximately 25–50 % of these macroadenomas can be defined as grossly invasive according to the most widely used radiological or surgical criteria (which are based on the degree of suprasellar extension or lateral extension into the cavernous sinus) [11–13, 15–21]. Around 50 % or more of tumors might be considered as invasive if microscopic invasion of the local dura is employed as the criterion [22, 23].

Several other clinically-relevant factors such as young age at diagnosis, large tumor size, and high GH secretion have also been identified as predictors of aggressive behavior [1]. Although pediatric cases of acromegaly are uncommon, these cases may present with more aggressive and larger tumors and, as such, may require more intensive management [24, 25].

## Resistance to therapy

As expected, the surgical cure rate is reduced for invasive tumors and many may be considered inoperable, thus necessitating other options, such as pharmacological intervention. In general, the surgical long-term remission rate for microadenomas is approximately 80 %, but this decreases to less than 50 % for macroadenomas and only 25–30 % for macroadenomas classified as grossly invasive [12, 13]. Extrasellar tumor growth, mixed tumors (GH- and prolactin-secreting), dural invasion, tumor size, cavernous sinus invasion, Knosp grade, and pre-operative GH and IGF-1 levels have all been shown to be predictors of poor surgical outcome [26, 27]. Persistent disease after surgery might require repeat surgery, which is indicated if there is residual tumor that is surgically accessible with a chance for surgical cure (or with persistent mass effects on the optic chiasm) [28], although pharmacological therapy is indicated if this is not the case.

Resistance to pharmacological therapy may be another manifestation of aggressive disease. In the case of somatostatin analogs (SSAs) many factors have been identified that predict a better response and some of these may be worth considering before commencing therapy [29]. For instance, older age and lower circulating GH levels at diagnosis have all been associated with better SSA response, and a positive acute SSA test (showing 75 % GH reduction) may also help to predict longer-term responsiveness [29, 30]. Other accessible measures, such as a hyperintense lesion on T2-weighted on MRI can predict a poor response, as can the presence of sparse GH tumor granulation histology [11, 29]. Various immunohistochemical assays for biochemical markers, such as Ki-67 LI (which is lower in responders), somatostatin receptor subtype 2 (SSTR2) expression, SSTR2/SSTR5 ratio and especially wild-type aryl hydrocarbon receptor-interacting protein (AIP) expression (which are all increased in responders) may also be useful for predicting response if biopsies are available. Recent data suggest that AIP can predict response to SSA therapy independently of SSTR2 expression, whereas the combination of both high AIP and high SSRT2 expression appears to be a particularly good predictor [31, 32]. Recent evidence also suggests that SSAs increase the expression of AIP and that it might play an important role in their mechanism of action [33]. However, these markers are not well validated in clinical practice and may not be widely available due to cost restrictions [29]. Nevertheless, if available, such factors may help to inform clinicians when making decisions about costly therapies.

The use of surgery to reduce tumor mass may help to improve the response to SSAs treatment in those resistant to primary SSAs therapy [34]. However, in those patients in whom all surgical and pharmacological approaches have failed to control the disease, radiotherapy is the only viable option [35, 36]. That said, novel pharmacological interventions are being investigated for the treatment of particularly aggressive pituitary tumors, most notably the orally administered alkylating agent temozolomide [37, 38]. However, experience with these agents specifically in patients with aggressive GH-secreting adenomas is extremely limited at present.

## Case study: difficult choices in a patient with invasive macroadenoma

- A 31-year-old man was referred by his cardiologist because of acral growth and deformity of facial features
- He was noted to have hypertension and impaired glucose tolerance 3-years earlier
- He also complained of fatigue and headache, arthralgia, carpal tunnel syndrome and erectile dysfunction
- Past medical history and familial history were unremarkable

Physical examination (initial evaluation)

- Clinical findings were hypertension, acral enlargement, prognathism, supracilliary arch prominence, macroglossia, gynecomastia and thickened skin
- No visual field defects

Hormonal evaluation

- GH nadir during OGTT = 17.3 μg/L [2-h glucose = 8.8 mmol/L (158 mg/dL)]
- IGF-1: 1,162 μg/L (normal 117–329 μg/L)
- T4: 103 nmol/L [8.0 μg/dL (normal 4.6–12 μg/dL)]
- TSH: 2.1 mIU/L (normal 0.3–4.1 mIU/L)
- Total testosterone: 5.5 nmol/L [159 ng/dL (normal 280–1,100 ng/dL)]
- Prolactin: 11.6 nmol/L [267 μg/L (normal 2–20 μg/L)]
- Basal cortisol/post Synacthen: 196/737 nmol/L (7/27 μg/dL)

Pre-treatment MRI

**Fig. 1:**
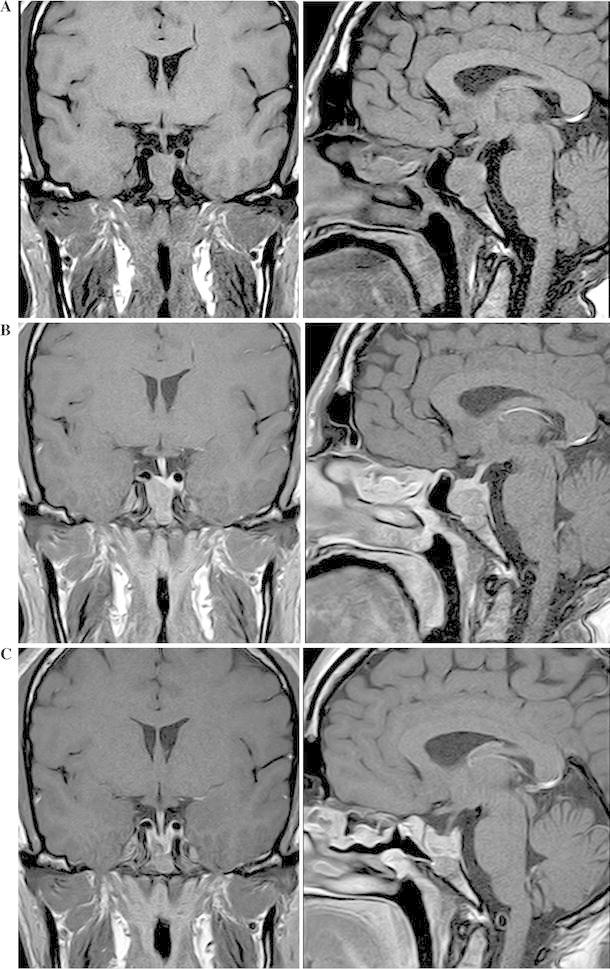
Case study: post-contrast, T1-weighted MRI scans of pituitary tumor at diagnosis (**a**), before surgery (**b**) and three months after surgery (**c**) (coronal* left*, sagittal* right*)

- Post-contrast coronal T1 MRI showed 18 × 13 mm macroadenoma in the right and inferior portion of the pituitary, with invasion of sphenoidal sinus and erosion of sphenoid and clivus. Tumor had hypointense signal and normal hypophysis showed homogeneous enhancement at left (Fig. 1a, b)

Diagnosis

- Acromegaly (with co-secretion of prolactin)
- Invasive macroadenoma without mass effect
- Hypogonadotrophic hypogonadism

Initial treatment

- Transsphenoidal surgery was performed (June 2009) in an attempt to improve the response to cabergoline or radiation therapy (SSAs were not included in medical insurance in Chile at the time of diagnosis)
- Histopathology evaluation showed positive staining for GH and prolactin, perinuclear cytokeratin staining (densely granulated) and Ki-67 LI 4.7 %
- Predictors of poor surgical outcome were extrasellar growth (clivus invasion) and mixed tumor

3-Months follow-up

- IGF-1 1193 μg/L (normal 115–307 μg/L)
- Random GH 10.2 μg/L
- GH nadir 8.6 μg/L
- MRI: 13 mm clivus residual tumor (Fig. 1c)

Subsequent treatment and course

- Cabergoline (3–5 mg weekly) was initiated in September 2009 without obtaining normalization of IGF-1
- Radiation therapy was performed in June 2010 without normalization of IGF-1
- In July 2010, SSAs were initiated as add-on therapy to cabergoline
- Biochemical cure was not achieved despite maximal doses of SSA
- Currently, random GH is 1.58 μg/L (ECLIA), IGF-1 650 μg/L, hypertension and impaired glucose tolerance are under control and no symptoms of obstructive sleep apnea are present. The patient complains of fatigue and joint pain

## Case discussion

This case provides an example of the difficult choices that have to be made in situations where access to some therapies is limited by local constraints, such as lack of reimbursement for specific drugs. The recommended first-line therapy in this patient was an SSA (possibly in combination with a dopamine agonist due to prolactin co-secretion). Due to the invasiveness of the tumor, the patient had a low chance of surgical cure. However, due to lack of access to SSAs at the time of diagnosis, a treatment plan had to be made based on the available local resources—in this case, surgery followed by cabergoline. Although the patient did not achieve biochemical remission even after SSAs became available, the patient has remained relatively stable. This introduces another dilemma regarding decisions to continue a pharmacological therapy or withdraw it and put the patient at risk of deterioration. Pressure to withdraw costly long-term pharmacological therapy may be considerable in areas with limited resources. Region-specific cost analyses would be useful to justify long-term pharmacological therapy in such situations. A further dilemma is whether to accept stable, but suboptimal, disease control or to intensify therapy further with additional cost. Guidelines recommend that patients should remain on the same dose for at least 3 months (assuming the patient tolerates the medication) to properly assess adequacy of treatment and the need for dose titration or switching to the GH receptor antagonist pegvisomant [39]. In the current case, intensification of pharmacological therapy beyond SSAs using the GH receptor antagonist pegvisomant was not possible, as it was not included in Chilean medical insurance programs.

## Conclusions

Choosing the correct individually tailored therapeutic option in a patient with acromegaly in line with current guidelines is a challenge, even when access to all available surgical, pharmacological and radiotherapeutical resources is available. This is particularly true in patients with more aggressive disease. Failure to achieve hormonal control in acromegaly puts patients at risk for early mortality, suggesting the need for aggressive efforts to normalize hormone levels [40]. In patients with aggressive, invasive tumors, access to all available options thus becomes paramount. The prognosis of surgery will depend on the characteristics of the tumor, but importantly, also on the experience of the neurosurgeon when it comes to treat patients with more invasive tumors [26].

The SSAs have a role as primary pharmacological therapy and as adjuvant therapy after surgery or radiation in patients with aggressive tumors. Newer drugs such as GH receptor antagonists can be effective in normalization of IGF-I in patients who do not respond to SSAs, but with a very high cost and access to these drugs may not be possible in many areas of Latin America [41, 42]. Combination therapy with SSAs and a GH receptor antagonist may be a safe and effective option that can help to lower doses and reduce the costs of pharmacological treatment [43–45]. Lower cost, but less effective, medications, such as dopamine agonists maybe the only pharmacological options available in areas with restricted resources. Fortunately, SSAs are becoming more widely available across Latin American countries and the use of pharmacological therapy has increased markedly over the last 10 years. Further development of the use of biomarkers to predict aggressive and invasive tumor behavior, as well as response to pharmacological therapy may help clinicians to make more informed treatment choices early and make more effective use of available resources.

In summary, in countries with less access to experienced pituitary surgeons and with limited access to all available pharmacological therapies, management of aggressive GH tumors is particularly complex and physicians may need to make difficult choices based on available resources. Methods to improve judicious treatment selection, such as the use of histological evaluation or development of new reliable and accessible biomarkers predicting response to therapy, may provide a means to optimize resource utilization.
